# A retrospective study of head and neck re-irradiation for patients with recurrent or second primary head and neck cancer: the McGill University experience

**DOI:** 10.1186/s40463-015-0084-4

**Published:** 2015-09-02

**Authors:** Rolina Al-Wassia, Siavosh Vakilian, Crystal Holly, Khalil Sultanem, George Shenouda

**Affiliations:** Department of Radiation Oncology, King Abdulaziz University, Abdullah Suleiman Street, P.O Box 80200, 21589 Jeddah, Saudi Arabia; Department of Radiation Oncology, McGill University Health Centre, McGill University, Montreal, Québec Canada; Department of Clinical Epidemiology, McGill University, Montreal, Québec Canada; Radiation Oncology, Segal Cancer Centre, Sir Mortimer B. Davis Jewish General Hospital, McGill University, Montreal, Québec Canada

**Keywords:** Re-irradiation, Head and neck, Squamous cell carcinoma

## Abstract

**Background:**

We report our experience with patients who received re-irradiation to the head and neck area for locoregional recurrences (LRR) or second primaries (SP) in a previously irradiated field.

**Methods:**

We reviewed 27 consecutive patients with a diagnosis of LRR or SP head and neck carcinoma treated with a second course of radiotherapy between April 2004 and July 2012. The main outcome measures were local control, overall survival, and complications. The results are expressed as actuarial values using the Kaplan–Meier estimates.

**Results:**

The median follow-up time was 24.7 months (range: 11 days–79.3 months). There were 23 males and four females with a median age of 61 years (range: 40–87 years). The actuarial overall survival rates at 1, 2, and 5 years were 77, 59, and 57 %, respectively. The actuarial local control rate was 80, 52, and 52 % at 1, 2, and 5 years, respectively. Three patients developed systemic metastases. The rate of grade 3 toxicity was 26 %, and that of grade 4 toxicity was 3 %. There were two treatment-related deaths (grade 5 toxicity).

**Conclusions:**

Continuous course re-irradiation in patients with LRR or SP head and neck cancer is feasible with acceptable toxicity. With current encouraging rates of local control and overall survival, this option should be discussed with patients who have few alternative therapeutic options.

## Background

Surgical resection is typically considered the modality of choice in patients with locoregional recurrences (LRR) or second primary (SP) head and neck cancer who were previously treated with a full dose of radiation therapy [1]. Historically, patients who were deemed to have unresectable tumors, because of tumor location, extent, or medical comorbidities, were referred for palliative chemotherapy. However, the response rates achieved with chemotherapy for these patients ranged between 10 and 40 % [2]. In the last decade, re-irradiation (RI) has begun to gain conceptual acceptance, as experimental and clinical studies have demonstrated that high-dose RI can be administered with reasonable success and acceptable complication rates.

The management of LRR or SP head and neck cancer in patients who were previously treated with a full dose of irradiation remains a clinical challenge. The difficulty arises from the possibility of serious side effects following RI [3, 4]. Some of these toxicities, such as carotid rupture, fistula, or bleeding, can be life-threatening. In addition, other serious but non-life-threatening side effects can occur – for example, osteonecrosis, soft tissue fibrosis, carotid stenosis, severe xerostomia, and trismus.

In spite of these complications, accumulated data from different centers [5–7] showed increased local control and survival in patients treated with a tri-modality approach, including surgery followed by RI and chemotherapy (if indicated), over single modality or chemotherapy alone.

Reasonable survival has been reported with primary RI alone, with a median survival of 10 months and a 3-year overall survival of 22 % [8, 9]. More commonly, however, chemotherapy is given concurrently to overcome radioresistance and to improve outcomes. The leading multicenter Radiation Therapy Oncology Group (RTOG 9610) trial examining concurrent RI and chemotherapy showed OS at 1 and 2 years of 40 and 15 %, respectively. In the other RTOG study (RTOG 9911), the OS rates at 1 and 2 years were 50.2 and 25.9 %, respectively. Both trials used a hyperfractionated, twice-daily RI schedule, to a total dose of 60 Gy in 1.5 Gy fractions. The improvement in outcomes in the second trial could be a result of using different chemotherapy agents, such as platinum-based regimens, which are known to be more effective for squamous cell carcinoma than hydroxyuria and 5-fluorouracil. More recently, Kharofa *et al.* [10] published encouraging results of their experience with a continuous course of RI and concurrent carboplatin and paclitaxel for locally recurrent squamous cell carcinoma of the head and neck. The authors reported a median survival of 16 months, and an OS of 54 % at 1 year and 31 % at 2 years.

The purpose of this study is to describe our institutional outcomes in comparison to other published data on RI among a similar group of patients.

## Methods

We retrospectively reviewed the medical records of 30 consecutive patients who received RI for either LRR or for in-field SP cancers between 2004 and 2012. Permission for data abstraction was obtained from the institutional Ethics Review Board. Three patients received brachytherapy as their RI modality and were excluded. Thus, 27 patients were included in the analysis.

### Patients

Patients included in this retrospective study were aged between 40 and 87 years at the time of the second diagnosis, with a median age of 61 years. There were 23 males (85 %) and four females (15 %). Twenty-six patients received RI to the head and neck area with curative intent, whereas one patient with metastatic disease at second presentation was re-irradiated with a palliative intent. The RI volume was delivered to overlapping areas that had previously been irradiated at the time of the first cancer diagnosis. All patients had histological proof of LRR or SP squamous cell carcinoma.

The diagnostic evaluation included a physical examination, panendoscopy with biopsies, radiologic evaluation of the head and neck by computed tomography (CT) and/or magnetic resonance imaging (MRI), and screening for distant metastases using CT and/or positron emission tomography.

For previously irradiated patients presenting with LRR or SP tumors, surgical salvage has remained the standard of care in our institution. In cases of unresectable lesions, primary RI, with or without concurrent chemotherapy, was discussed with the multidisciplinary tumor board and, if deemed appropriate, the option was presented to the patient. Only patients with good performance status (Eastern Cooperative Oncology Group [ECOG] performance status of ≤2) were considered candidates for RI.

Postoperative RI was considered only if the pathological features of the surgical specimen indicated a high risk of subsequent recurrence [11, 12], such as positive margins, lymph node metastasis with extracapsular extension, and/or multiple lymph node metastases.

### Tumor

Primary head and neck tumor sites and the initial stage of disease are reported in Table 1. The recurrence was defined as local if the tumor recurred in the primary site in the previous radiation field, regional if it recurred in the previous radiation field but outside the primary site, and locoregional if the tumor recurred in both the primary site and in the regional nodes. After the first course of radiotherapy, 11 patients (41 %) had failed locally, four (15 %) had failed regionally, 10 (37 %) had failed locoregionally, and two had SP (7 %).

**Table 1:** Patient and tumor characteristics at first presentation

	Number	Percent
Patient	27	
Male	23	85
Female	4	15
Median age	61	
Tumor site at first presentation		
Larynx	5	18
Oropharynx	7	26
Nasopharynx	7	26
Maxillary sinus	1	4
Nasal cavity	1	4
Oral cavity	2	7
Unknown primary	1	4
Hypopharynx	1	4
Esophagus	2	7
Histology		
Squamous cell carcinoma	25	92
Undifferentiated	2	8
Stage at first presentation		
TxN0M0	1	4
T1-4N0M0	8	30
T1-4N1M0	7	26
Tx-4N2M0	9	33
Unknown	2	7

For tumor classification, the sixth edition of the Union Internationale Contre le Cancer (UICC) was used. Detailed information on staging is shown in Table 1. RI was not necessarily given at the time of the second diagnosis; in five patients, RI was given at the third diagnosis, as radiotherapy was not indicated in either the first or second courses of treatment. Of these five patients, four had recurrences and one patient had a SP.

Salvage surgery was performed in 12 patients (44 %) before RI and resulted in clear margins in four cases, close or positive margins in five cases, and gross residual disease in three cases. Concurrent chemotherapy or targeted therapy during RI was given in 21 patients (77 %) at the discretion of the treating physician, and it included various agents (cisplatin, carboplatin, 5-fluorouracil, or cetuximab). Six patients received RI alone.

### Treatment

In our present study, two different schedules for RI were used: one was similar to the RTOG bid schedule mentioned above, and the second consisted of a total dose of 60 Gy in 30 fractions once daily. Radiotherapy was given concurrently with chemotherapy, usually consisting of a platinum-based regimen, although targeted therapy such as cetuximab was also used; this was similar to other reports in the literature [13]. Radiotherapy was given with 4–6 MV photon linear accelerators using a head and neck thermoplastic immobilization mask. Treatment was given using either three-dimensional conformal radiotherapy, the intensity modulated radiotherapy (IMRT) technique, or Helical TomoTherapy, depending on the available resources at each of the two treatment sites. From 2004–2006, IMRT was mainly used (15 patients), whereas starting from 2007, 11 patients were treated with Helical TomoTherapy. The remaining patient received three-dimensional conformal radiotherapy.

The gross tumor volume (GTV) was defined as any macroscopically visible disease, detected by radiological investigations or by clinical exam, in both the primary tumor and the lymph nodes. A maximum margin of 1 cm was applied to the GTV to define the expansion to clinical target volume (CTV). The CTV to planning target volume margin was 5 mm in three-dimensional conformal radiotherapy and IMRT patients, and only 3 mm in TomoTherapy because of the image-guided radiotherapy function that allowed better day-to-day reproducibility of patient positioning. There was no attempt to treat any elective lymph node area or other areas at risk outside the CTV volume.

**Fig. 1:**
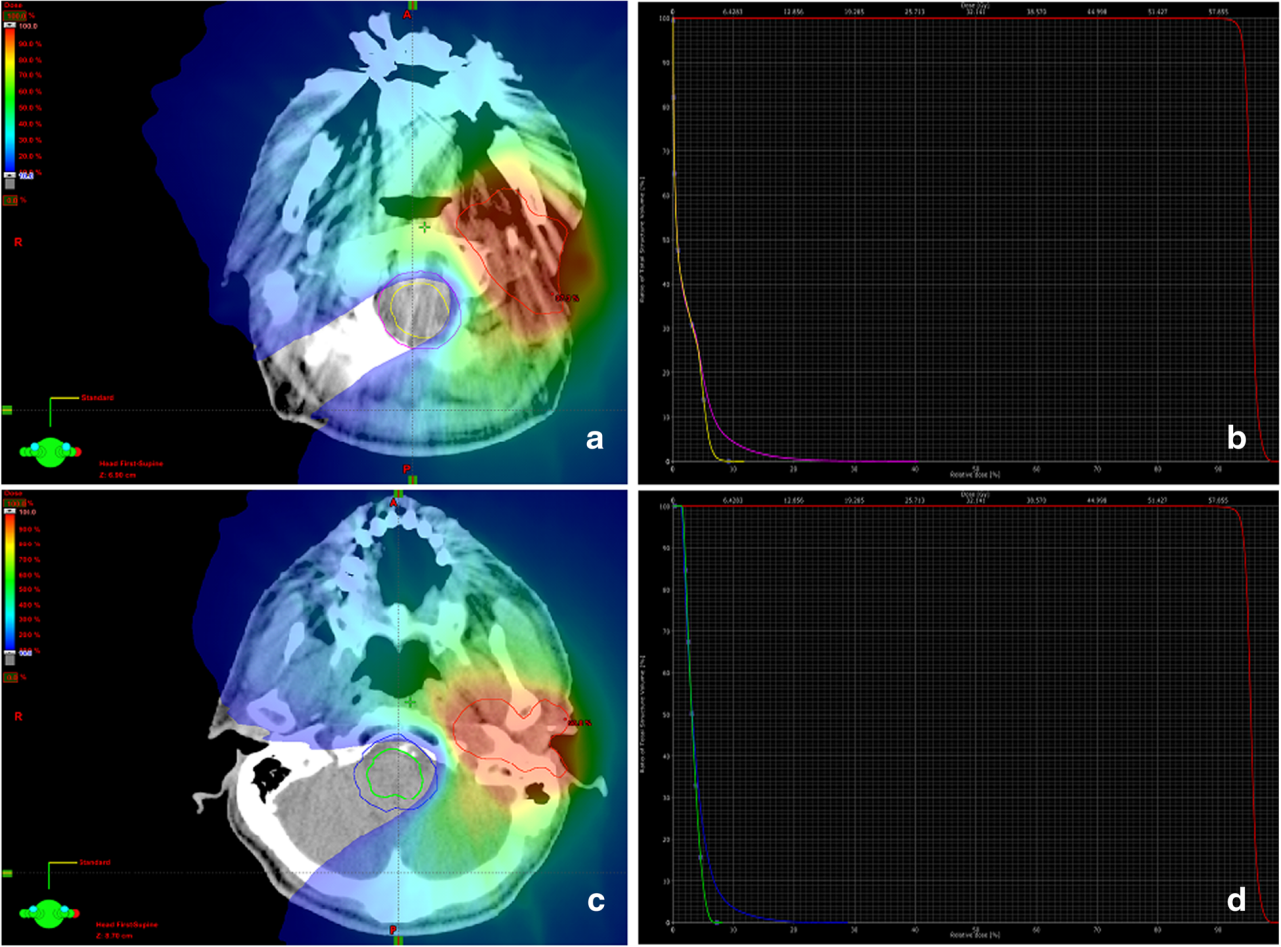
**a** Color wash dose distribution and **b** dose volume histogram showing spinal cord and PRV sparing, **c** color wash dose distribution, and **d** dose volume histogram showing brainstem and PRV sparing. *PRV* planning organ at risk volume

The most important organs at risk when RI was considered were the spinal cord, brainstem, salivary glands, optic apparatus, and mandible. For the spinal cord and brainstem, the dose was also calculated to a planning organ at risk volume (PRV), which was created by adding a 5 mm three-dimensional margin to the organ at risk. We limited the maximal spinal cord dose at retreatment to 20 Gy, with a maximum PRV dose of 22 Gy; a maximal dose to the brainstem of 20 Gy, with a maximum PRV dose of 22 Gy; a mandible dose of 40 Gy to <50 % of its volume; and 50 % of the parotids and salivary glands would receive no more than 25–30 Gy. Cumulative lifetime doses after RI were measured for all patients for whom complete information on the first treatment was available. Two patients received their first radiation treatment outside Canada, which precluded the calculation of cumulative doses to target volumes. Figure 1 illustrates how modern techniques such as IMRT allowed for excellent target coverage, while meeting strict constraints on the organs at risk, such as the brainstem and spinal cord.

In our series, RI was indicated in different clinical settings: as primary definitive treatment in 14 patients; as adjuvant treatment postoperatively in 12 patients; and as palliative treatment in one patient, as shown in Table 2.

**Table 2:** Treatment characteristics at the time of RI

	Number	Percent
Surgery		
Postoperative RI + systemic therapy	9	33
Postoperative RI alone	3	11
Definitive RI without surgery		
RI + systemic therapy	12	45
RI alone	2	7
Palliative RI	1	4
Concurrent chemotherapy/targeted therapy		
Cisplatin-based	19	70
Cetuximab	2	7
None	6	22

*Abbreviation*: *RI* re-irradiation

### Statistics

All statistical analyses were performed using SPSS software version 10.0 (SPSS Inc, Chicago, IL, USA). Results were expressed as actuarial values using the Kaplan–Meier estimates. Actuarial and median survivals were calculated from the first day of the RI course.

## Results

From 2004–2012, 27 patients with LRR or SP head and neck cancer received RI at our institution. The median follow-up time was 24.7 months (range: 11 days–79.3 months).

The median maximal dose delivered to the spinal cord at retreatment was 15.5 Gy (range: 6–45 Gy), the median maximal dose delivered to the brainstem was 20 Gy (range: 1–63 Gy), and the median dose to the mandible was 63 Gy (range: 5–75 Gy). It is noteworthy to realize that these numbers represent median values of the maximal doses, which are often received by a very small volume of the irradiated organ. For both parotids, the mean dose was 28 Gy (range: 1–72 Gy).

### Disease control

**Fig. 2:**
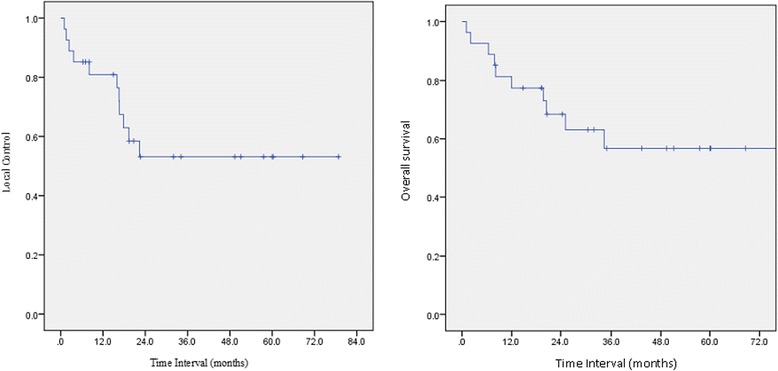
Local control and overall survival

The actuarial estimates of local control were 80, 52, and 52 % at 1, 2, and 5 years, as shown in Fig. 2. The median time to the first recurrence or the SP was 24.5 months (range: 4.6–283 months). The median time to the third diagnosis or second failure was 17 months (range: 3.5–192 months).

Response to treatment after the second course of radiation was measured on either CT or MRI. The maximal radiation response was judged 6 months after the completion of radiation therapy. Nineteen patients (70 %) had a complete response, four patients (15 %) had a partial response, one patient (4 %) had no response, two patients (7 %) had progression of disease, and one patient had insufficient follow-up to evaluate response to treatment.

At a median follow-up of 24.7 months, 14 patients (52 %) had no evidence of failure, four patients (15 %) had local failure, three (11 %) had regional failure, two (7 %) had locoregional failure, two (7 %) had SP, and two (7 %) had persistent disease. Five patients received their second course of radiation at the third diagnosis. Two of these patients were diagnosed with a SP, while the other three had local failures. Four out of these five patients had received high-dose radiotherapy. All four remained locally controlled. The remaining patient with esophageal cancer had received a palliative dose and had immediate local progression; the patient died shortly after.

### Overall survival and distant metastasis

The actuarial OS rates at 1, 2, and 5 years were 77, 59, and 57 %, respectively (Fig. 2), calculated from the first day of the RI course. At a median follow-up of 24.7 months, 17 patients (64 %) were alive. Only three patients (11 %) developed systemic metastases; one patient developed metastasis and died during the treatment course.

### Toxicity

The National Cancer Institute Common Toxicity Criteria for Adverse Events version 3.0 (CTCAE) was used for toxicity grading. Overall late grade 1–3 toxicity was reported in 25 (93 %) of the treated patients. Details of toxicity are presented in Tables 3 and 4. Two grade 5 toxicities occurred: one as a result of carotid rupture leading to death, and one death secondary to mucosal bleeding (in a patient with locally recurrent disease). No brainstem or spinal cord injuries or brain necrosis were observed.

**Table 3:** Late toxicities

Toxicity	Grade 1	Grade 2	Grade 3	Grade 4	Grade 5
Dry mouth	21 %	52 %	7 %		
Dysphagia	29 %	36 %	11 %		
Trismus	28 %	31 %	7 %		
Muscle fibrosis	25 %	25 %	11 %		
Vascular					7 %
Loss of taste	43 %	11 %			
Hearing loss	7 %	21 %	3 %		
Radio-osteonecrosis		7 %	7 %	3 %

**Table 4:** Grade 4 and 5 severe adverse events

Primary site	Primary stage	Initial treatment	Primary response	Second diagnosis	Interval between first and second diagnosis (months)	Life time accumulated dose	Toxicity description	Interval between second radiation and toxicity (months)
Oropharynx cancer	T4N2b	Concurrent Platinum-based chemotherapy + radiation 70/35 Gy	Complete	Local recurrence	17.7	134 Gy	Carotid rupture	6 months
							Causing death	
Maxillary cancer	T3N2	Radiation 74/32 Gy	Complete	Second primary, oropharynx cancer	51.8	144 Gy	1 – osteonecrosis, fractured mandible, disabling; 2 – skin fistula with bone exposed to air	24 months
Oropharynx cancer	T2N1	Surgical resection followed by adjuvant radiation of 60 Gy	Complete	Locoregional recurrence	29	120 Gy	Mucositis, 5 × 6 cm ulceration causing bleeding, leading to death	5 months

## Discussion

In our group of patients receiving high-dose RI for head and neck LRR or SP tumors, we found excellent actuarial local control of 52 % and OS of 57 % at 5 years. These compare favorably with findings from the reported literature. The report from the University of Texas MD Anderson Cancer Center [14] showed a median time to progression of 7 months and progression-free rates at 1, 2, and 5 years of 44, 34, and 29 %, respectively. The median OS was 16 months, and the OS at 1, 3, and 5 years was 54, 31, and 20 %, respectively. Sher *et al.* [15] reported the results of 35 patients with recurrent head and neck cancer treated with continuous course RI, while using platinum-based chemotherapy and an IMRT technique. The actuarial 2-year survival was 48 %, with a 2-year locoregional control rate of 67 % and a median OS of 1.9 years. Lee *et al.* [16] reported the Memorial Sloan–Kettering Cancer Center’s experience of 105 patients with recurrent head and neck cancer who underwent RI with chemotherapy in 75 % of patients. An IMRT technique was used in 70 % of patients. The 2-year locoregional progression-free and OS rates were 42 and 37 %, respectively.

A few reasons could account for our good results. Unlike some other studies [5, 7], all but one of our patients received some form of IMRT, either on a linear accelerator or on a Helical TomoTherapy unit. This has allowed for the delivery of RI in a more conformal fashion, minimizing acute toxicities and thus reducing treatment interruptions. The fact that our long-term toxicity data compare favorably with those of the reported literature (only three grade 4 or 5 toxicities, despite the relatively common grade 3 toxicities) again reinforces the positive effect of IMRT techniques employed in our group of patients, allowing for the delivery of radical doses of RI to tumor-bearing volumes, with significant sparing of the critical normal previously irradiated organs. Also, 78 % of patients in the current report received some form of concomitant systemic therapy, most of them with cisplatin. Our results can also be attributed, at least partially, to careful patient selection. In the current series, one-third of the patients had node-negative disease at first presentation, and almost one-half had recurrences that were only local, without lymph node involvement, and all patients had an ECOG of ≤2.

The current series is limited by the relatively small sample size of 27 patients. Also, given the different practices at our two affiliated institutions, our patients did not all receive the same dose/fractionation schedule. Fifteen patients were treated to 60 Gy in 2 Gy fractions once daily; however, a significant minority (six patients) received twice-daily fractionation. In addition, 12 % of patients did not receive concurrent systemic treatment. On the other hand, irradiation techniques were homogeneous, with all but one patient receiving IMRT.

In the current series, the planning target volume based on the GTV was re-irradiated with no attempts to treat any elective nodal sites. This approach was similar to that of previous series that reported on their experiences from different centers [17–20], and which showed that the majority of failures after RI were local at the site of the recurrent GTVs (rGTV). In the Michigan series [21], where RI included the rGTV with no elective neck nodal irradiation, the authors studied 66 patients at a median follow-up of 42 months and found that all LRRs occurred within the rGTVs except for two (4 %). Sher *et al.* [15] reported that 73 % of LRRs occurred within the RI volumes in patients treated with an IMRT technique to the rGTV alone. In the series by Popovtzer *et al.*, [21], 71 % of patients had presented with evidence of local failure after RI, while neck-only failures occurred in two patients (5 %). These results confirm that recurrent local disease continues to be a significant challenge in patients with RI for LRR or SP tumors in the head and neck region.

Most of the reported series currently tend to use a continuous RI course using once-daily fractionation schedules [22–24]. A recent report from the Beth Israel Medical Center was published on the use of Intra-Operative-Radiotherapy (IORT) in patients with loco-regional recurrent head and neck cancer. Seventy-six patients were identified who underwent treatment to a total of 87 sites after gross-total resection. The 2-year estimate loco-regional control was 62 % with a median survival of 19 months and a 2-year survival rate of 42 %. The authors concluded that IORT was well tolerated and was associated with an encouraging local-regional disease control and an improved overall survival [25].

## Conclusions

In conclusion, our results reinforce the emerging view in the scientific community that RI with concomitant chemotherapy for LRR or SPs, in a region that previously received high-dose irradiation, is feasible, and it produces good local control with chances of long-term survival; it also features acceptable, albeit not negligible, long-term toxicity. More importantly, clinical judgment and careful patient selection, as well as the judicious use of modern IMRT/image-guided radiotherapy techniques are critical components for the safe delivery of RI. The care of these patients requiring RI to the head and neck region is complex and should be carried out by centers where necessary multidisciplinary expertise and support are available.

## Abbreviations

CT: Computed tomography
CTV: Clinical target volume
ECOG: Eastern Cooperative Oncology Group
GTV: Gross tumor volume
LRR: Locoregional recurrences
MRI: Magnetic resonance imaging
OS: Overall survival
PRV: Planning organ at risk volume
rGTV: recurrent gross tumor volume
RI: Re-irradiation
SP: Second primary
